# Current Trends in Development of Liposomes for Targeting Bacterial Biofilms

**DOI:** 10.3390/pharmaceutics8020018

**Published:** 2016-05-24

**Authors:** Zora Rukavina, Željka Vanić

**Affiliations:** Department of Pharmaceutical Technology, Faculty of Pharmacy and Biochemistry, University of Zagreb, A. Kovačića 1, 10000 Zagreb, Croatia; zpalac@pharma.hr

**Keywords:** liposomes, biofilm, antimicrobial, infection, drug delivery, physicochemical properties

## Abstract

Biofilm targeting represents a great challenge for effective antimicrobial therapy. Increased biofilm resistance, even with the elevated concentrations of very potent antimicrobial agents, often leads to failed therapeutic outcome. Application of biocompatible nanomicrobials, particularly liposomally-associated nanomicrobials, presents a promising approach for improved drug delivery to bacterial cells and biofilms. Versatile manipulations of liposomal physicochemical properties, such as the bilayer composition, membrane fluidity, size, surface charge and coating, enable development of liposomes with desired pharmacokinetic and pharmacodynamic profiles. This review attempts to provide an unbiased overview of investigations of liposomes destined to treat bacterial biofilms. Different strategies including the recent advancements in liposomal design aiming at eradication of existing biofilms and prevention of biofilm formation, as well as respective limitations, are discussed in more details.

## 1. Introduction

Biofilms are defined as immobilized microbial colonies interspersed and encased within a self-produced matrix of hydrated extracellular polymeric substances (polysaccharides, proteins, nucleic acids and lipid molecules). Complex and heterogeneous structure of extracellular matrix is transected with network of water and nutrient channels, which are necessary for the exchange of nutrients, water and metabolic products among cells. The exact structure and composition of biofilms are influenced by numerous factors including surface properties, nutrient availability and microbial constituents of the biofilm [1].

**Figure 1:**
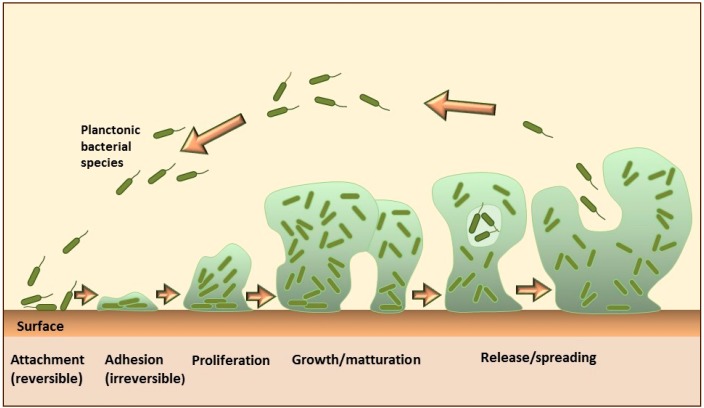
Schematic drawing of biofilm formation (adapted from [6,7]).

The formation of the biofilm on solid surfaces is a step-wise process comprising several stages (Figure 1). It starts with the conditioning of the surface through the coating with macromolecules from the aqueous surrounding, which enables initial reversible adhesion of microorganisms. The next step is a formation of stronger, irreversible attachments to the surface, followed by the proliferation and aggregation of microorganisms into multicellular and multilayered clusters, which actively produce extracellular matrix. Some cells in the mature biofilms continuously detach and separate from the aggregates, representing a continuous source of planktonic bacteria that can subsequently spread and form new microcolonies [2]. Both the growth and the maturation into a differentiated biofilm, as well as maintenance of such highly organized communities are regulated through the intercellular communication via molecular chemical signals, also known as quorum sensing (QS) [3]. QS affects the cell density, gene expression, resistance to stress, virulence factor production and other cellular functions in the biofilm [4,5].

Biofilms are a common cause of chronic, nosocomial and medical device-related infections, due to the fact that they can develop either on vital or necrotic tissue as well as on the inert surfaces of different implanted materials. Moreover, biofilms are linked with high-level resistance to antimicrobials, frequent treatment failures, increased morbidity and mortality. As a consequence, biofilm infections and accompanying diseases have become a major health concern and a serious challenge for both modern medicine and pharmacy. The rough estimation shows that more than 60% of hospital-associated infections are attributable to the biofilms formed on indwelling medical devices, which result in more than one million cases of infected patients annually and more than $1 billion of hospitalization costs per year in the USA [7,8]. For example, the catheter related infections and sepsis alone result in approximately 900,000 admissions per year in the USA, which cost additional $28,000 per case [7]. Taking the aforementioned into account, as well as an increasing number of implantable medical devices available and the constant aging of the population, it is rather clear that the infections associated with biofilms represent a heavy and expanding economic burden for the healthcare systems worldwide. Apart from the medical device-related infections (prosthetic joints, prosthetic heart valves, central venous and urinary catheters, intrauterine devices, contact lenses), there are numerous medical conditions in humans that can also be associated to biofilm appearance such as recurrent urinary infections and prostatitis [9], slow- and non-healing chronic wounds and burns [10], periodontitis [11], chronic pulmonary infections in cystic fibrosis (CF) [12], chronic otitis media [13], chronic rhinosinusitis [14], native valve endocarditis [2,15] and others.

Biofilm infections share some common characteristics: slow development in one or more hot-spots, delayed clinical manifestation, persistency for months or years, usually with interchanging periods of acute exacerbations and absence of clinical symptoms [16]. Even though they are less aggressive than acute infections, their treatment is challenging to a greater extent. The main reason for the aforesaid is up to 1000-fold decrease in susceptibility of biofilms to antimicrobial agents and disinfectants as well as resistance to host immune response [5]. As reported in many reviews, the increased biofilm resistance is hypothesized to be a result of various possible mechanisms [5,17,18,19,20,21]. For instance, increased tolerance could be partly explained by the fact that the biofilm-constituting microorganisms are embedded within extracellular polymeric glycocalix, mostly anionic in nature, which represents certain penetration impediment, probably through slowing and delaying diffusion or by chemical and electrostatic interactions with antimicrobial molecules [17,21]. Chemical interactions involve enzymatic degradation of antibiotics, especially since the extracellular matrix provides appropriate environment for the accumulation of high amounts of antibiotic-inactivating enzymes within biofilm. Another proposed theory for elevated biofilm resistance is that the specific mosaic structure of biofilm generates gradients of oxygen, nutrients, pH-value, ionic strength and redox potential throughout the biofilm, which lead to corresponding heterogeneity in growth rate and metabolic activity of biofilm residents in different layers [22]. Due to changes in proliferation speed and gene transcription microorganisms in biofilms also manifest altered phenotype as compared to planktonic cells. The mutation and horizontal gene transmission frequency in biofilms is significantly higher as compared to their planktonic counterparts, which could explain why biofilm-growing microorganisms easily become multidrug resistant [23]. All of these proposed mechanisms contribute to the lack of successful treatment and result in re-infection due to incomplete biofilm eradication. The majority of the proposed biofilm-control methods focuses on: (i) prevention and minimization of biofilm formation by selection and surface modifications of anti-adhesive materials [24,25]; (ii) debridement techniques including ultrasound and surgical procedures [26,27,28]; (iii) disruption of biofilm QS-signaling system [4,29]; or (iv) achieving proper drug penetration and delivery to formed biofilms by the use of electromagnetic field [30], ultrasound waves [31], photodynamic activation [32] or specific drug delivery systems [18,33].

Among different drug delivery technologies that have been explored and developed for treatment of the biofilm-related infections nanotechnology plays an important role. During the last few decades a variety of nano-scaled drug delivery systems have been designed and employed, including polymeric nanoparticles, dendrimers, metal nanoparticles, solid lipid nanoparticles and liposomes [7,8,34,35,36,37]. These nanosystems are considered a promising strategy for overcoming biofilm resistance as they enable improved delivery of antimicrobials to bacterial cells thus increasing the efficacy of treatments. Lipid-based vesicular nanosystems are particularly interesting due to the safety issue and targeting ability.

This review provides an overview of the different types of liposome-based drug delivery (nano)systems, currently investigated for prevention and/or eradication of bacterial biofilms. They are classified according to the bilayer composition, membrane rigidity/elasticity, surface properties and abilities to trigger the release of the encapsulated antimicrobials. Special emphasis has been given to the novel approaches in liposome-based biofilm delivery and the impact of the physicochemical properties influencing the efficacy of the formulations.

## 2. Liposomes for Targeting Bacterial Biofilms

### 2.1. General Considerations

Liposomes are physiologically acceptable (phospho)lipid-based vesicles composed of one or more lipid bilayers enclosing inner aqueous compartment(s). Such a structure facilitates encapsulation of a wide variety of drugs/active ingredients; hydrophilic into the inner water space, lipophilic inside bilayer and amphiphilic between these two regions. Liposomes are characterized by their phospholipid composition, morphology, particle size, surface properties and membrane rigidity/elasticity, parameters that determine their stability and interactions with biological milieu [38,39,40]. Many reviews report liposomes as the most widely used antimicrobial drug delivery nanosystem [7,34,35,36,37,41,42,43]. Moreover, they have been explored to be applied via different routes of drug administration including parenteral [35], dermal [40,44], vaginal [45,46], pulmonary [47,48] and ocular [49] route. Liposomes are expected to increase the bioavailability, biocompatibility and safety profiles of encapsulated drug [40]. Their unique feature lies in lipid bilayer structure mimicking cell membranes and allowing fusion with bacterial membranes. Consequently, the entrapped drug can be released to the cell membranes or the interior of the microorganisms [50].

### 2.2. The Role of Liposomal Physicochemical Properties

**Figure 2:**
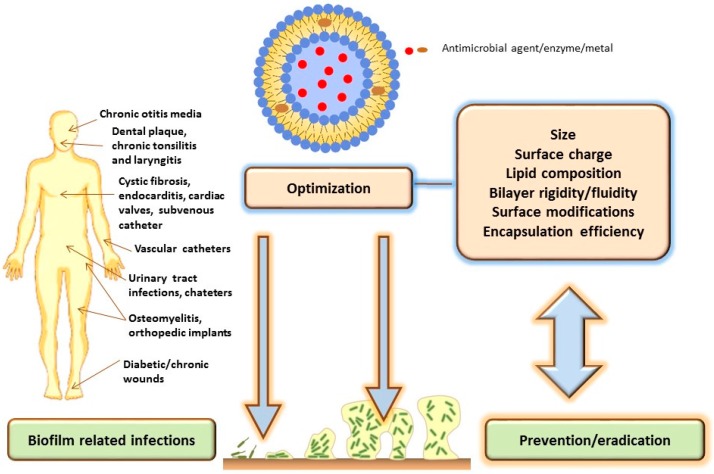
Influence of the liposomal physicochemical properties on the biofilm delivery.

Physicochemical features of liposomes have been proven to influence their efficacy in prevention and eradication of biofilms. Appropriate size, narrow size distribution, suitable bilayer features, surface characteristics and high encapsulation efficiency are of great importance for effective antimicrobial delivery to biofilms (Figure 2). In addition, biofilm properties including the complex composition of extracellular matrix and physicochemical characteristics of antimicrobial agents (drugs, enzymes, metals) should also be considered during design of an effective liposome formulation [7,42].

The concept of non-specific targeting using the liposomes with the surface charge opposite to the surface charge of bacteria enables an extended contact time of antibiotic with biofilms. Robison and collaborators have shown that each bacterium in biofilm adsorbed independently and that the extent of adsorption was significantly decreased for anionic liposomes in comparison to positively charged vesicles of the same size range [51]. Cationic formulations (both conventional and fusogenic liposomes) were thus more effective against negatively charged bacteria due to their ionic interaction and fusion with the bacterial cell envelope [52]. Moreover, the size of liposomes and the thermodynamic state of the bilayers were found to be responsible for stability of the vesicles during storage, release pattern as well as interaction with biofilm and bacteria [42]. Thus, the majority of all studied conventional liposomes that were effective in delivery of antimicrobials to biofilms had a size significantly below 500 nm and consisted of the relatively rigid lipid bilayers. Namely, the bilayers composed of saturated phospholipids with longer fatty acid chains and higher ratio of cholesterol, contribute a higher degree of membrane stability (reduced leakage) as a consequence of suppressing the gel to liquid-crystalline transition. This increases the possibility of the liposomes to release encapsulated antimicrobials at the target site (on the surface or inside the extracellular matrix of biofilms). Although the interaction between liposomes and biofilms differed for each species of biofilm, generally, the smaller and positively charged liposomes penetrated better into the biofilms (extracellular matrix) than anionic liposomes of the same size range [53]. Another important parameter that could also determine the efficacy in biofilm delivery is the concentration of liposome formulation applied including the lipid concentration and the concentration of the encapsulated antimicrobial agent [54].

The modifications of the liposome surface by covalently attached antibodies (immunoliposomes) [55] or lectins, such as Concanavalin A [56], allowed specific targeting to biofilms. The enhanced antibacterial activity obtained by immunoliposomes was due to increased retention of the immunoliposomes in *Streptococcus oralis* biofilm facilitating release of antibiotic in close proximity of the bacteria during extended period of time [55]. The mechanism of liposome targeting by lectin functionalized liposome lies in selective binding of Concavalin-A to α-mannopyranosyl and α-glucopyranosyl residues that can be found in the extracellular polysaccharide matrix of some biofilms [57]. The efficacy of functionalized liposomes was found to be dependent on the liposome composition and surface density of Concavalin-A affecting biofilm binding affinity. Thus increasing the ratio of negatively charged phosphatidylinositol (PI) reduced the efficacy of dipalmitoyolphosphatidylcholine (DPPC)/PI/dipalmitoylphosphatidylethanolamine (DPPE) liposomes to *Streptococcus mutans* biofilm [56].

Apart from the liposome characteristics, anti-biofilm activity is also determined by the physicochemical properties of entrapped antimicrobial and its mechanism of action. Different drugs encapsulated in the liposomes of the same physicochemical properties have demonstrated dissimilarities in the extent of antibacterial action. While both liposomal ciprofloxacin and meropenem displayed bactericidal activity at or below minimal inhibitory concentrations (MIC) for the free drugs, liposomal gentamicin exhibited higher MIC values compared to the free antibiotic. Meropenem is amphiphilic drug that can easily penetrate the bacterial outer membrane, while ionic interaction between the liposome and bacterial surface ensures that a high concentration of meropenem is delivered directly into the periplasm at concentration below the MIC for the free drug. On the other hand, gentamicin is hydrosoluble and binds to 30S subunit of bacterial ribosomes, preventing protein synthesis. Before gentamicin is transported to cytoplasm it must first bind ionically with the bacterial cell surface, an interaction that is dependent on the composition and structure of the lipopolysaccharide layer. The encapsulated drug is unable to interact in this way, and is instead reliant on interaction of the liposomes in which it is entrapped [42,58].

Various liposomal formulations differing in the physicochemical features (Table 1) have been explored to increase delivery of antimicrobials to biofilms and biofilm-associated microorganisms. They are classified in several groups according to their design and physicochemical characteristics, as thoroughly discussed in the following sections (Section 2.3, Section 2.4, Section 2.5, Section 2.6, Section 2.7, Section 2.8, Section 2.9 and Section 2.10).

### 2.3. Conventional Liposomes

This term denotes classical (plain) liposomes, without surface modifications, composed of phospholipids with or without addition of cholesterol. According to the surface charge achieved by the addition of charged lipids into bilayers they are categorized as neutral, anionic or cationic liposomes. Cationic (positively charged) liposomes have shown great potential for biofilm targeting due to their interaction with the negatively charged biofilm surface. One of the first articles by Jones group dealt with the adsorption of stearylamine containing liposomes to *Staphyloccocus epidermidis* biofilms. The extent of adsorption was found to be dependent on the liposomal lipid concentration and was described by Langmuir adsorption isotherm. The strong affinity of cationic liposomes for *S. epidermidis* was found to be of an electrostatic nature and involved negative charges associated with the bacterial biofilm [59]. This phenomenon was dependent on the ionic strength of the surrounding medium, whereas by increasing the ionic strength the biofilm-vesicle dissociation decreased [60]. To overcome the toxicity related to stearylamine, dimethyldioctadecylammonium bromide (DDAB) has been used as an alternative cationic lipid for the preparation of vancomycin liposomes. Compared to neutral liposomes, which also displayed some inhibiting effect on the growth of *S. epidermidis*, the inhibition was more pronounced for cationic liposomes due to their biofilm adsorption ability. Namely, uncharged liposomes affected the bacteria by releasing hydrophilic vancomycin into the surrounding medium, thus allowing the drug to come into the contact with the biofilm. Besides the surface charge, antibacterial activity was also affected by entrapment efficiency of vancomycin in liposomes. The smaller number of liposomes with higher encapsulation efficacy inhibited the growth more than the larger number of liposomes with relatively low efficacy of the encapsulated drug. In comparison to liposomal vancomycin, cationic liposomes with gentamycin were less effective probably as a result of high molecular weight of gentamycin affecting the slow passage through the membrane [61]. Further study performed on *Staphylococcus aureus* biofilms has also shown positive results, whereas DDAB-based cationic liposomes with vancomycin inhibited bacterial growth from the biofilms and were more effective than the equivalent amount of the free antibiotic for short (30 min) liposome-biofilm incubation times [62]. Both anionic and cationic liposomes were superior in delivery of triclosan and chlorhexidine as compared with the free bactericides. Double labelling experiments using [14C]-chlorhexidine and [3H]-DPPC suggested diffusion mechanism being responsible for bactericide delivery [63]. Continued research on mixed bacterial biofilms (*Streptococcus salivarius* DBD and *Streptococcus sanguis* C104) has demonstrated a linear relationship between the liposome adsorption and biofilm composition for both cationic and anionic liposomes whereas the extent of adsorption was smaller for anionic liposomes. Surprisingly, anionic liposomes with triclosan were the most effective in inhibiting growth of *S. sanguis* C104 biofilms, whereas growth of *S. salivarius* DBD could not be effectively diminished by liposomal triclosan [51]. An incorporation of 22 mol% dimethylammonium ethane carbamoyl cholesterol (DC-Chol) as cationic lipid into the lipid bilayers composed of dipalmitoylphosphatidylcholine (DPPC) and cholesterol (Chol) was shown to enhance delivery of encapsulated benzyl penicillin G to *S. aureus* biofilms. The time to reach maximum growth rate from biofilms was investigated as a function of overall drug concentration ranging from 2.9 × 10^−3^ mM to 1.09 mM, and as a function of time of exposure to liposomal drug in a range of 1.5 s to 2 h. The effectiveness was greater at the lower overall drug concentrations tested and the shorter time of exposure of the liposomes to the biofilm, as compared to the free antibiotic. It was demonstrated that the time to reach maximum growth rate from *S. aureus* biofilms could be extended by a factor 4 relative to the free drug [64].

Summarizing all of the above reported studies on conventional liposomes, it is evident that vesicles with relatively rigid bilayers have been used and the size of all the vesicles examined was always less than 200 nm. However, none of these early investigations involved *in vitro* toxicity assessments and/or animal studies to approve biocompatibility and efficacy of developed formulations.

Several research groups have evaluated the potentials of conventional liposomes in eradication of *Pseudomonas aeruginosa* (the major pathogen in the lungs of CF patients, which grows in small colonies with biofilm-like characteristics) in *in vivo* studies (animals) and clinical trials. Thus, Meers *et al.* have investigated biofilm penetration potential, mechanism of drug release and *in vivo* antimicrobial activity of amikacin loaded neutral DPPC/Chol liposomes designed for nebulization and inhaled delivery. To visualize the penetration into the biofilm, fluorescently labeled liposomes have been prepared and monitored either by the filter assay and epifluorescence or the confocal scanning laser microscopy. Vesicles with a mean diameter of approximately 300 nm penetrated readily into *P. aeruginosa* biofilm and infected mucus, whereas vesicles larger than 1 µm failed to do so. Biofilm penetration and targeted, sustained release of amikacin, mediated by sputum or rhamnolipides in biofilm supernatants, resulted in superior *in vivo* efficacy of inhaled liposomal amikacin in comparison to the free drug formulation. Namely, rhamnolipids in the *P. aeruginosa* biofilms and CF sputum, due to surfactant-like characteristics, trigger the release of the amikacin from DPPC/Chol liposomes. Because the rhamnolipid concentration is higher in the close proximity of biofilm, liposomes release the antibiotic at the site of infection [65]. The superiority of the liposomal amikacin (Arikace™) for the therapy of bacterial biofilm infections has been confirmed in the clinical studies. Arikace™ passed the Phases II and III of clinical trials in CF patients with *P. aeruginosa* infections proving the tolerability, safety, biologic activity and efficacy [7,66,67,68]. Besides Arikace™, liposomal ciprofloxacin (Lipoquin™) has also been developed for the therapy of *P. aeruginosa* infections in CF patients. Lipoquin™ contains ciprofloxacin loaded hydrogenated soy phosphatidylcholine (HSPC)/Chol (7:3, weight ratio) liposomes, approximately 90 nm in diameter. Safety and efficacy of the Lipoquin™ has been confirmed in a 14-day Phase II trial on 22 adult CF patients, where statistically significant reduction of *P. aeruginosa* colonization and increase in the lung function were achieved [69]. Deeper insights on the inhaled liposomal ciprofloxacin formulations are provided in the very recent review by Cipolla and collaborators [70].

Alhajan *et al.* have examined the efficacy and safety aspects of liposomal clarithromycin in the treatment of *P. aeruginosa* biofilm infection. The drug has been encapsulated in neutral (DPPC/Chol), anionic (DPPC/Chol/dicethyphosphate) and cationic (DPPC/Chol/DDAB) liposomes of approximately 200 nm in diameter. The results of microbiological evaluation confirmed complete eradication of *P. aeruginosa* biofilm with both positively and negatively charged liposomes whereas the effect was more pronounced with cationic liposomes. All of the tested liposomal formulations were less toxic than the free drug as shown on a pulmonary epithelial cell line (A549). Amongst various liposome formulations explored, anionic liposomes were selected as optimal formulation due to the highest encapsulation efficiency, biofilm effect and safety profile achieved [71].

Negatively charged and neutral liposomes with tobramycin have been examined for eradication of *Burkholderia cepacia* complex biofilm. Unfortunately, the anti-biofilm effect of both types of liposomes was not increased in comparison to the free drug. Electrostatic repulsive forces between the bacterial surface and anionic liposomes as well as the low encapsulation of tobramycin in neutral liposomes were suggested to be responsible for obtained results [54].

Recent study by Omri group reports on the activity of neutral (DPPC/Chol) liposomes with azithromycin against biofilm-forming *P. aeruginosa*. MIC and minimal bactericide concentrations (MBC) with the liposomal formulation were significantly lower than those obtained with the free drug. Liposomal azithromycin significantly reduced the growth of bacteria in the biofilm and attenuated the production of different virulence factors. Complete eradication of bacteria in biofilm was achieved at concentration of 512 mg/L, while the fusion between bacterial membrane and liposomes was responsible for the increased antimicrobial effect. The safety of liposomal azithromycin has been proven through the toxicity evaluation on the A549 human lung cells and hemolytic test [72].

### 2.4. Fusogenic Liposomes

Fusogenic liposomes, also known as Fluidosomes™, have been designed to improve the delivery of antimicrobials by facilitating fusion of liposomal and bacterial membranes. The bilayers of these liposomes contain optimized portion of lipids causing the disturbances in the packing of phospholipid bilayer. Namely, the presence of asymmetric lipid (phosphatidylethanolamine) or lipids with shorter acyl chains and increased number of double bounds induce the lowering of the membrane transition temperature or disorders in lipid packing [7,73]. In comparison to the conventional liposomes typically composed of the rigid membranes, the fusogenic liposomes are characterized by relatively fluid (soft) lipid bilayers. Pioneering research on fusogenic liposomes by Beaulac *et al.* has demonstrated an increased *in vivo* bactericidal activity of tobramycin encapsulated in the negatively charged fluid liposomes. The vesicles composed of DPPC and dimiristoylphosphatidylcholine (DMPG) (10:1 and 15:1, molar ratios) with Tc of 29.5 and 33 °C were effective in the treatment of chronic pulmonary *P. aeruginosa* infection in comparison to distearoylphosphatidylcholine (DSPC)/dimyristoylphosphatidylcholine (DMPG) liposomes (10:1 or 15:1 molar ratios) with Tc of 40 and 44 °C, respectively. More than 27 µg tobramycin/mg of tissue has been determined in the lungs of rats 16 h after the last treatment with DPPC/DMPG (15:1) liposomes. The superiority of the formulation has been confirmed by increased localized effect, whereas significantly lower levels of the drug were detected in kidneys than with the free drug [74]. It should be noted that Fluidosomes™ have been verified as non-immunogenic and can be administered repeatedly without adverse immune responses to control chronic pulmonary infections [75]. Except for *P. aeruginosa*, the antimicrobial activity of tobramycin Fluidosomes™ has been confirmed against biofilms of *Stenotrophomonas maltophilia*, *B. cepacia*, *S. aureus* and *Echerichia coli*. The sub-MICs of tobramycin liposomes reduced the growth of *P. aeruginosa*, *B. Cepacia*, *S. maltophilia*, *E. coli* and *S. aureus* by factors 84, 129, 166, 105 and 104, respectively, as compared to the free drug [76]. The increased penetration of the antibiotic in the Gram-negative bacterial cells has been attained with dry powder preparations of tobramycin Fluidosomes™ as well. Sixteen hours after the treatment with the powdered liposomes the growth of *P. aeruginosa*, *S. maltophilia*, *B. Cepacia* and *E. coli* in the presence of sub-MICs of tobramycin was significantly lowered by 17, 40, 47 and 50 times, respectively, in comparison to the free drug. However, the overall efficiency was greater with liquid Fluidosomes™ due to the lower drug encapsulation in dehydrated Fluidosomes™ and the circumstance that the powdered liposomes were rehydrated directly in the medium culture at 37 °C instead at 63 °C [77]. The fact that sub-MICs of encapsulated antibiotic reduced the bacterial growth by 35–105 times in comparison to cultures treated with the same quantity of the free antibiotic suggests a direct interaction of liposomes with bacteria [76].

Various techniques including negative staining, immunoelectron microscopy, fluorescence activated cell sorting and lipid-mixing studies have confirmed fusion between Fluidosomes™ and bacterial cells leading to a marked increase of tobramycin in a cytoplasm of resistant *P. aeruginosa* strains. The time needed to reach the maximal fusion was approximately 5 h for resistant strain compared to a much shorter time for the sensitive strain. These specific properties of fusogenic liposomes are valuable for overcoming bacterial resistance related to permeability barrier of *P. aeruginosa* [50]. The superiority of tobramycin fusogenic liposomes has been proven *in vivo* on a rat model of chronic lung infection with *B. cepacia* where intratracheal administration markedly changed its pulmonary pharmacokinetic profile, resulting in a slower distribution and a slower elimination [78]. To the best of our knowledge, tobramycin Fluidosomes™ is currently ongoing Phase II clinical study in Europe [79].

Besides using phospholipids with shorter fatty acid chains and increased number of double bounds, fusogenic liposome can be also prepared by incorporating asymmetric lipids like 1, 2-dioleoyl-*sn*-glycero-2-phosphoethanolamine (DOPE) in the phospholipid bilayer. The encapsulation of vancomycin into DPPC:DOPE:cholesteryl hemisuccinate (CHEMS) liposomes extended the spectrum of vancomycin action against the drug resistant *E. coli*, *Klebsiella* spp., *P. aeruginosa* and *Acinobacter baumannii*. The enhanced penetration of the drug has been achieved through the fusion of liposomal- with the outer- membranes of the Gram-negative bacteria. MICs ranging from 6 (*E. coli* and *A. baumannii*) up to 83.7 mg/L (*P. aeruginosa* ATCC27853) were determined for fusogenic liposomes, while neither the conventional liposomes nor free vancomycin showed any activity [80].

Drulis-Kawa *et al.* have evaluated *in vitro* activity of meropenem encapsulated in various fluid and rigid liposome formulations against *P. aeruginosa* strains that are meropenem-resistant due to low permeability or production of carbapenemases. The highest bactericidal activities were achieved with cationic fluid liposomes composed of phosphatidylcholine (PC), DOPE and stearylamine (4:4:2, molar ratio), and rigid liposomes consisting of PC, 1,2-oleoyloxy-3-trimethylammonium-propane (DOTAP) and Chol (5:2:3, molar ratio). Their MICs for the meropenem-sensitive strains were 2–4-fold lower than MIC of the free drug. However, none of the studied liposomal formulations exhibited bactericidal activity against drug-resistant isolates. Surprisingly, even Fluidosomes™ (DPPC/DMPG liposomes, 18:1), showed 4–16-fold higher MICs for resistant strains than did free meropenem. These results were explained by the differences in the composition of the outer membranes of resistant and susceptible strains [81], meaning that surface characteristics of each strain have to be taken into consideration during the design of an effective liposome formulation.

In addition to fusogenic liposomes discussed above, another type of liposomes with flexible (elastic) liposomal membranes has been proposed for biofilm targeting. Daptomycin containing liposomes composed of lecithin and sodium cholate (17:1, mass ratio) have been proven to efficiently diffuse into the skin showing rapid and efficient antibacterial activity against *S. aureus*. Effective therapeutic concentrations were maintained for several hours and significantly inhibited bacterial growth and injury-induced biofilms. Although the mechanism of action has not been evaluated, the authors hypothesized that flexible liposomes may facilitate greater penetration of daptomycin into the biofilm [82].

### 2.5. Surface-Modified Liposomes

One of the strategies proposed to combat biofilm related infections is the use of surface-modified liposomes such as poly(ethylene) glycol (PEG)-derived liposomes (PEGylated liposomes), immunoliposomes, lectin-coated liposomes and mannosylated liposomes.

Steric stabilization of liposomes via PEGylation is a common approach in designing long-circulating liposomes for parenteral drug delivery. PEGylated liposomes containing antibiotics have also been considered for the treatment of bacterial biofilms. Ahmed and collaborators have tested the efficacy of different surface charged PEGylated liposomes (both anionic and cationic), approximately 120 nm-sized, in combating *S. aureus* biofilms. Adsorption of liposomes to immobilized biofilms of *S. aureus* was found to follow Langmuir isotherm, which assumes coverage of the biofilm by a monolayer of liposomes, where each binding site is being identical and independent. However, the affinity of liposomes for the biofilms was inhibited by PEGylation, suggesting that the steric stabilization of liposomes would only prolong the circulation time of liposomes in the blood, without improving the delivery into the biofilms [84]. Contrary to these findings, Moghadash-Sharif *et al.* have demonstrated that PEGylation of rifampin-loaded cationic liposomes, having the same size as the corresponding liposomes in the previous study, have not reduced the ability of liposomes in eradication of *S. epidermidis* biofilm. The affinity of these liposomes to bacteria was not changed and the formulation was as effective as non-PEGylated cationic liposomal formulation. In addition, the incubation time was found to play an important role in the efficacy of liposomes, whereas the increasing of incubation time has led to enhanced biofilm eradication [85].

Liposomes can be specifically targeted to oral bacteria by the use of monoclonal antibodies. The potential of immunoliposomes in reducing dental plaque related to *S. oralis* biofilm has been reported by Robinson and coworkers. The targeting affinity of the immunoliposomes to *S. oralis* biofilm was found to be largely unaffected by the number of antibodies conjugated to the liposomal surface or by the net charge of the liposomal lipid bilayer. In comparison to the liposomes of the same lipid composition but without the antibody (“naked” liposomes, *i.e.*, conventional anionic liposomes), the biofilm affinity of anti-oralis immunoliposomes was significantly higher, but was still lower than affinity of the free antibody. Interestingly, when their effect has been compared with cationic liposomes incorporating stearylamine, adsorption of the immunoliposomes to *S. oralis* biofilm was lower than with the conventional cationic vesicles [86]. Continuing the research with triclosan and chlorhexidine loaded anti-oralis immunoliposomes the same group of investigators has proven improved growth inhibition of *S. oralis* biofilms using the low bactericide concentrations. Several times increased inhibition effect with anti-oralis immunoliposomes was found, for short exposure times to the biofilms, compared to the free bactericides. The extent of the growth inhibition by the antibacterial immunoliposomes was linearly related to the number of immunoliposomes targeted to the biofilm surface [55].

Vyas *et al.* have shown that coating of metronidazole liposomes composed of PC and stearylamine with lectin (Concanavalin-A) inhibited the growth of *S. mutans*, the Gram-negative bacteria that harbor in the periodontal pocket (biofilm), during six hours of incubation. In comparison with the other studies where nm-sized liposomes were examined, the average size of lectinized liposomes was 2.9 µm. The lectinized vesicles exhibiting the zeta potential of −5 mV maintained the sugar affinity and specificity of the associated ligand, and were able to target the surface glycocalyx of the bacterial biofilm. Moreover, lectin-coated liposomes were found to be stable in the simulated salivary fluids (pH 7.2) and under various pH conditions [56].

Effective targeting to the bacterial biofilm could also be achieved by mannosylated liposomes. Unlike the PEGylation where the surface of the vesicles is hydrophilic, mannosylation is related to anchoring of liposomes with hydrophobic mannan derivatives such as cholesteryl mannan and sialo-mannan. Although both types of mannosylated PC/Chol liposomes with metronidazole exhibited increased activity against *S. aureus* biofilm comparing to the non-mannosylated liposomes, the superior targeting ability was achieved with sialo-mannan vesicles. The improved antimicrobial activity with mannosylated liposomes was probably a result of their ability to adsorb or fuse with the bacterial biofilm due to ligand-mediated interactions and subsequent release of all or a part of the antimicrobial agent in the proximity of the target [88].

### 2.6. Reactive Liposomes Encapsulating Enzyme(s)

Liposome encapsulation of singular or coupled enzyme system(s) represents a promising approach for biofilm prevention/eradication. Namely, these enzymes, after exposure to the certain substrate, produce the hydrogen peroxide and/or succeeding oxidizing agents that exhibit broad-spectrum antimicrobial activities. Moreover, liposome usage ensures adsorption and retention of the encapsulated enzymes in the close proximity to the biofilm surface.

Glucose oxidase (GO) and coupled enzyme system glucose oxidase-horse radish peroxidase (GO-HRP) were encapsulated in DPPC/PI liposomes for targeting the oral *Streptococcus gordonii* biofilms. Glucose was used as a primary substrate for GO, resulting in the production of hydrogen peroxide, antibacterial agent itself, which in the presence of iodide and HRP enzyme yields oxyacids (hypoiodus, iodic and periodic acid), displaying antibacterial activities as well. Comparison of the activity between GO- and GO-HRP-reactive liposomes have demonstrated that the coupled enzyme system is more effective in inhibiting growth for shorter times of exposure of the *S. gordonii* biofilms to the liposomes [89]. This approach has been extended to enzymes chloroperoxidase and lactoperoxidase in combination with GO, using the same phospholipids for the enzyme encapsulation and targeting of *S. gordonii* biofilms. These reactive liposomes were shown to be effective in inhibiting the growth of tested biofilms, even in the presence of saliva, which is important in the context of their potential application in the oral cavity [90]. Both studies have proven that the antibacterial activity of reactive liposomes encapsulating enzymes was proportionally dependent on the enzyme content in the vesicles, liposomes-biofilm incubation time and exposure time of liposomes-biofilm system to the enzyme substrates [89,90]. The growth inhibition for all of the tested formulations was significantly higher when the incubation time was prolonged from 1 to 30 min, approaching almost 70% of inhibition. However, from the practical standpoint considering the delivery systems applicable to the oral cavity, it is highly advantageous to achieve activity after a brief exposure. One way to overcome this limitation would be repetitive application [90].

Numerous parameters affect the production of bactericidal species by reactive liposomes such as enzyme encapsulation efficiency, the permeability of the liposomal bilayer to substrate and the bactericides produced, the temperature and the liposomal bilayer composition. Kaszuba and Jones have investigated these factors with respect to the production of hydrogen peroxide from anionic and cationic liposomes with incorporated GO and peroxidases in the presence of glucose substrate. The production of hydrogen peroxide by the encapsulated enzymes was increased almost linearly with the corresponding concentration of the encapsulated GO and with the substrate concentration. Depending on the bilayer lipid composition, liposomal encapsulation of the enzymes had the opposite effect on the bilayer permeability for the substrate analogue. Whereas for the anionic (DMPC/PI) liposomes an increase in the permeability was detected, the permeability was reduced for the cationic (DMPC/DDAB) liposomes, which could be related to the ionic and hydrophobic interactions of the negatively charged enzymes with charged lipid constituents [106].

### 2.7. Antibiotic-Metal Co-Encapsulating Liposomes

Liposome co-encapsulation of antibiotics with particular metals has been proposed as the novel nanoencapsulation strategy to improve their antimicrobial and anti-biofilm activities. For instance, gallium is a transition metal known to inhibit bacterial growth and biofilm formation through disruption of iron metabolism and iron-dependent cellular processes [107,108,109,110]. Following this approach, DPPC/dipalmitoylphosphatidylglycerol (DPPG) liposomes encapsulating both gallium and gentamicin (Lipo-Ga-GEN) were developed to combat clinical isolates of *P. aeruginosa*. The formulation was shown to be more effective than liposome loaded gentamicin and the free antibiotic against both planktonic and biofilm growing *P. aeruginosa*. Moreover, full eradication of the bacterial biofilm as well as the interruption of QS signaling was achieved with Lipo-Ga-GEN, while the toxicity of gallium was significantly reduced [91]. The same group has also worked on the analogous therapeutic approach for the treatment of infections associated with CF, based on the co-encapsulation of tobramycin and bismuth-ethanedithiol (BiEDT) within DSPC/Chol liposomes (LipoBiEDT-TOB) [92,93]. Similarly as gallium, bismuth and bismuth-thiol have demonstrated promising antimicrobial effects against a wide range of bacteria, by affecting iron-uptake, alginate expression, lipopolysaccharides, and secretion of virulence factors, bacterial adherence and biofilm formation [111,112,113,114,115]. The experiments with LipoBiEDT-TOB have shown superior bactericidal activity against several non-mucoid and mucoid strains of *B. cepacia* and *P. aeruginosa*. Significantly lower MIC and MBC values were achieved than with the free drug and the drug free Lipo-BiEDT, respectively. In comparison to both the free BiEDT and tobramycin, LipoBiEDT-TOB resulted in the significantly reduced cellular toxicity, as determined on A549 human lung cancer cells [92]. LipoBiEDT-TOB has also been proven to possess QS suppressing properties at concentrations below the MICc for the free drug and bismuth, respectively, and inhibited biofilm forming *P. aeruginosa* growth at 0.064 mg/L. None of the LipoBiEDT-TOB system components separately (BiEDT, tobramycin, BiEDT-tobramycin, liposomal tobramycin, liposomal BiEDT) displayed better activity. In addition, the formulation has been shown to penetrate sputum samples collected from CF patients and was not affected by the presence of polyanions typical to CF lungs [93]. These findings were further expanded in the works of Alipour and coworkers [94,95]. As measured by the changes in QS, virulence factors, twitching and attachment of *P. aeruginosa*, synergistic effect of tobramycin and bismuth was achieved with LipoBiEDT-TOB. The liposomal formulation exhibited comparable effects to the free agents, but at 4–16-fold lower concentrations. Moreover, the results attained by continuous culture flow cell analyses have demonstrated the potential of LipoBiEDT-TOB to penetrate deeper into the biofilm than free BiEDT-TOB, enabling killing of bacteria not only on the surface but also in the core of biofilm complex [95]. Continuing the research on activity of the liposomal formulation in combating *P. aeruginosa* biofilm, Alipour *et al.* have found that LipoBiEDT-TOB in synergy with alginate lyase increased the susceptibility of the mucoid biofilm, and attenuated the alginate production, which is the major contributor to *P. aeruginosa* resistance [94]. Although the described antibiotic-metal co-encapsulating liposomes have shown encouraging results *in vitro*, they were to a certain extent supported *in vivo*. Intratracheal administration of LipoBiEDT-TOB to rats chronically infected with *P. aeruginosa* showed a 4.3-log reduction in colony forming units (CFU) *versus* a 2.7-log reduction in CFU with free BiEDT-TOB. However, despite the considerable reduction of bacterial counts in the lungs, the complete eradication of bacteria was not accomplished after three days of treatment. It was hypothesized that the reason could be the high stability of vesicles composed of DSPC and cholesterol which limits the release of tobramycin at adequate concentration to ensure complete eradication and the negative contribution of agar beads used to induce infection in the experiments [96].

Recently, the anti-biofilm activity of liposome-entrapped combinations of BiEDT or bismuth-propanedithiol with imipenem, ciprofloxacin and ceftazidime, have been evaluated. The optimal concentrations of bismuth-thiols used with the antibiotics were found to have predominantly synergistic inhibitory effects on *P. aeruginosa* biofilms, with the exception of combining BiEDT with imipenem and ceftazimide [116].

### 2.8. Liposomes-in-Hydrogel

This approach comprises application of antibiotic loaded liposomes embedded in a suitable base (e.g., hydrogel), wherein antimicrobial effect of liposomal drug could be further improved by the right choice of gel base. Apart from increased viscosity of liposome formulations, incorporation of liposomes into semisolid bases enhances the retention of liposomes at the site of application and can contribute to improved physical stability of liposomes, drug release properties and potentially penetration of liposomes and encapsulated drug [45,117,118,119]. Some vehicles such as chitosan hydrogel, even without incorporated drug, revealed certain antimicrobial effect and have been proven to disrupt bacterial biofilms [120]. In addition, the bioadhesive properties of chitosan hydrogels [117], as well as ability to promote drainage and prevent increase of exudates in wound therapy [121], make them very promising for the topical treatment of burn wounds. Recently, Hurler and colleagues have explored the potential of mupirocin liposomes-in-chitosan hydrogel for the healing of burn wounds in mice model. The formulation was shown to be non-toxic against keratinocytes but able to prevent the formation of *S. aureus* biofilm. However, the antimicrobial activity was more pronounced against planktonic bacteria than against mature biofilms. *In vivo* investigation of liposomal mupirocin-in-chitosan hydrogel on healing of burden wounds demonstrated the efficacy of formulation comparable to the marketed product of mupirocin. Moreover, the healing time for the liposomal hydrogel was shorter in comparison to the marketed cream [103].

DiTizio *et al.* have tested the ability of ciprofloxacin loaded liposomes incorporated into cross-linked poly(ethylene glycol)-gelatin hydrogel to prevent bacterial adhesion and biofilm formation on urinary catheters. Liposomal hydrogel-coated catheters contained 185 µg cm^−2^ of the antibiotic that was gradually released over seven days permitting complete inhibition of bacterial adhesion. The additional value of the developed formulation was in improved biocompatibility of coated catheters since lubricious hydrogels tend to minimize the tissue inflammation associated with the other types of catheters [104]. *In vivo* evaluation of catheters coated with ciprofloxacin liposomal hydrogel proved effective delay of the development of catheter-associated bacteriuria in rabbits. The time to bacteriuria detection in 50% of the specimens was double from ciprofloxacin liposomal hydrogel-coated catheters in comparison to untreated and the drug free liposomal hydrogel [105].

### 2.9. Solid Supported Liposomes (SSLs) and Liposome Loaded Scaffolds (LLSs)

Solid supported liposomes (SSLs) are combined delivery systems based on drug loaded liposomes adsorbed onto the surface of solid particles, such as zinc citrate or calcium sulfate.

Catuogno and Jones have designed SSLs encapsulating triclosan or penicillin G to target immobilized biofilms of *S. oralis* and to exploit possible and desirable synergistic effect of the liposome-encapsulated bactericides with zinc citrate [97], which itself is used in dental preparations to prevent bacterial growth and plaque accumulation [122]. The adsorption of anionic (DPPC/PI) and cationic (DPPC/DDAB/Chol) liposomes to *S. oralis* biofilms was found to be dependent on the lipid concentration, whereas the optimum was achieved at 19 mol% of PI or DDAB. Similarly, the biofilm targeting with the drug loaded SSLs was found to be dependent on the amount of the lipid adsorbed onto zinc citrate particles. Free triclosan and penicillin G were shown to be effective against *S. oralis* growth when used in concentration ≥125 µg/mL (each bactericide), while the zinc citrate particles showed antibacterial properties only at the high concentration tested (5%). Interestingly, solid zinc citrate particles in combination with the free drugs displayed regressive effects as compared to the separate antibacterial activity of the each component. The tested SSLs showed no particular advancement in the antibacterial activity in comparison to the activity of the individual constituents, while addition of the drug or liposomes to the solid particle dispersions inhibited the bactericidal activity of the particles [97].

In another research, gentamicin loaded DPPC/SA/Chol liposomes (1:0.49:0.43, molar ratio) impregnated onto the calcium sulfate particles (CS) were examined for the treatment of chronic staphylococcal osteomyelitis [98]. Contrary to the above discussed study [97], the impregnation of gentamicin liposomes onto the CS particles proved to have no regressive effect on the anti-biofilm activity of the liposomal gentamicin, since *in vitro* anti-biofilm activity of gentamicin SSL was found to be the same as of the non-adsorbed liposomal gentamicin. *In vivo* study on rabbits revealed that gentamicin SSLs were significantly more effective than both gentamicin-impregnated CS and non-adsorbed liposomal gentamicin. The complete sterilization of bone tissues was achieved in the animals treated with the SSLs. Moreover, implantation of the antibiotic loaded SSL was found to be more appropriate for the treatment of bacterial osteomyelitis than the conventional injection of the free drug due to improved delivery to the infection site [98].

Apart from antibiotic loaded SSLs, biofilm targeting can be also achieved by liposome-loaded scaffolds (LLSs), *i.e.*, liposomally-entrapped antibiotics incorporated onto the artificial, porous and biodegradable bone scaffolds. Several investigations have demonstrated the potentials of LLSs to combat *S. aureus* biofilm occurrence in the osteomyelitis [99,100].

Zhu *et al.* have loaded various gentamicin-sulfate liposomes, differing in the size (0.1–5 µm), into the pores and onto the surface of beta-tricalcium phosphate granules (0.5 mm). Release kinetic of the antibiotic from the LLS has shown initial rapid release of liposomal gentamicin from the scaffold matrix followed by the sustained release of the free drug from liposomes. The anti-biofilm properties of LLS with gentamicin content ranging from 2.5–800 µg/mL were investigated *in vitro* on *S. aureus* biofilms. When compared to the control (free antibiotic solution of equivalent concentrations), gentamicin containing LLS demonstrated significantly elevated anti-biofilm activity. Bacterial re-growth in the anti-biofilm experiments was shown to be influenced by the liposome particle size in addition to the drug entrapment efficiency. Thus LLS with the smallest liposome size of approximately 100 nm, at the lowest drug concentration (2.5 µg/mL), showed the highest inhibiting effect. However, at the higher drug concentration range (400–800 µg/mL) the maximum antibacterial activity was achieved with the liposomes in size range of around 850 nm [99].

Ma and collaborators have investigated a suitability of a nano-hydroxyapatite/chitosan/konjac glucomannan scaffold (n-HA/CS/KGM) for the incorporation of cationic vancomycin loaded liposomes [100]. The n-HA/CS/KGM is biodegradable and biocompatible scaffold with the possibility of regulating drug release through the adjustment of the chitosan/konjac glucomannan (CS/KGM) ratio, while the content of hydroxyapatite (n-HA) can be optimized to imitate natural bone [123]. The tested n-HA/CS/KGM scaffold contained 60%–70% of n-HA, whereas the content of KGM and impregnated cationic liposomes varied to investigate potential influence on release properties. When the amount of KGM in the scaffold was increased, slower release of the drug has been confirmed, probably as a result of the swelling tendency of the KGM, which could facilitate sustained release in clinical application. Anti-biofilm effects were determined *in vitro* for blank, free vancomycin loaded scaffold and vancomycin LLS. The drug LLS more successfully inhibited the formation of *S. aureus* biofilms than the free drug loaded scaffold, especially at the lower drug concentrations and shorter exposure time. Interestingly, blank scaffold itself also reduced slightly the biofilm formation [100], probably due to the presence of chitosan, which is known to have antibacterial activity [124,125].

In the following study [101], nano-hydroxyapatite/beta-tricalcium phosphate scaffold loaded with cationic liposomal ceftazimide has been evaluated by the same group of authors. *In vitro* release profile of ceftazimide from ceftazimide LLS showed the initial burst release in the first several hours and nearly 100% release within 72 h. Liposomal ceftazimide inhibited *S. aureus* biofilm formation more efficaciously than the free drug and the drug LLS in the first 2 h of exposure. However, during the longer incubation time (2–12 h), ceftazimide LLS exhibited the most prominent anti-biofilm activity [101].

Summarizing *in vitro* and *in vivo* results from all of the above-discussed LLS-based investigations, it is obvious that LLSs could offer a new platform in development of clinically applicable delivery systems for the local anti-biofilm therapy of osteomyelitis.

### 2.10. Miscellaneous

An interesting approach for the treatment of device-associated osteomyelitis has been proposed by Liu and coworkers. It relies on the use of biomineral-binding liposomes (BBLs) to enable rapid delivery of encapsulated antimicrobials onto the orthopedic device surface. For this purpose an alendronate-tri(ethyleneglycol)-cholesterol conjugate (ALN-TEG-Chol) was incorporated into the lipid bilayers during the preparation of oxacillin containing liposomes. The presence of ALN-TEG-Chol in the liposome bilayers induced the significant changes in the thermodynamic behavior of liposomes, but was also expected to yield anchoring of the BBLs to the surface of hydroxyapatite coated orthopedic implants. The results of anti-biofilm testing against *S. aureus* biofilm have shown significantly higher inhibitory effects of oxacillin BBLs than that of the non-binding liposomes, suggesting that the BBLs could be utilized against *S. aureus* colonization and biofilm formation in the prevention of orthopedic device-related osteomyelitis [102].

To improve prevention of dental infections (caries) associated with insoluble glucan-biofilm synthesis, Yamakami and collaborators suggested liposomal encapsulation of peptide antibiotic nisin. Liposomes consisting of hydrogenated lipids (Phospholipon 90) and phytosphingozine (2:0.4, molar ratio) have been shown to effectively inhibit glucan-biofilm synthesis by *S. mutans*. The concentration of nisin-liposome required for the successful inhibition of glucan-biofilm synthesis was four-fold lower than that of naked nisin following 2 h cultivation. Namely, liposome formulation prolonged the inhibitory activity of nisin against glucan-biofilm synthesis by *S. mutans* 10449 for up to 6 h, while naked nisin gradually lost this inhibitory activity over the same period [126].

## 3. Toxicity Aspects

Majority of the aforementioned investigations have dealt with liposomal delivery to biofilm *in vitro*, whilst keeping toxicity studies towards representative mammalian cell lines to a minimum. However, these studies are crucial to prove the biocompatibility of formulations and usually precede the *in vivo* animal and clinical investigations.

Liposomes are commonly recognized as biocompatible, biodegradable and relatively non-toxic as they are composed of lipids from natural sources that reduce the toxicity of entrapped antimicrobial agents. For instance, free bismuth-ethanedithiol (BiEDT) at concentration of 10–20 µM rendered A549 human lung cancer cells non-viable. On the other hand exposure of A549 human lung cancer cells to the liposomal tobramycin co-encapsulating BiEDT reduced its toxicity. In all cases, liposomal encapsulation preserved the cell viability in comparison to cells treated with free bismuth, tobramycin or their combinations with empty liposomes [92].

Considering the influence of the physicochemical properties of liposomes on the cell viability, it has been proven that both surface charge and lipid concentration affect biocompatibility of liposomes [36]. In general, positively charged liposomes composed of cationic lipids have shown cytotoxic effects while negatively charged and neutral were less toxic or without toxicity effects to the treated cells [71]. Another important issue that should be kept in mind relates to the organic solvents used for the liposome preparation, particularly for liposomes destined for parenteral delivery. Namely, residues of toxic organic solvent might not be completely removed from the final liposome dispersion, causing a high risk of cytotoxicity via different mechanisms including the enzyme inhibition, protein denaturation, cell membrane modification and extraction of certain cellular components (lipids, cholesterol, and protein) [36]. Such unfavorable effects can be controlled by ensuring complete removal of residual solvents by applicable methods [127].

The long-term toxicity/safety issue of liposome-based drug delivery systems especially those for parenteral administration and treatment of chronic infections has to be determined. The potential long term toxicity of repeatedly administered cationic liposomes remains an issue that needs to be addressed [128].

## 4. Limitations of Liposomal Formulations

Despite the numerous advantages, liposomal antibiotics exhibit some drawbacks such as the limited stability of the vesicles and low entrapment of hydrophilic drugs. The stability of liposomes is determined by their lipid compositions with pH and storage temperature as prevailing parameters [35]. The stability of liposomes can be improved by addition of antioxidants to prevent oxidation of phospholipids, while hydrolysis can be hampered by preparing liposome dispersions at neutral pH, storage at lower temperatures and by freeze-drying of liquid liposome formulations [127,129]. Another problem associated with physical instability is the drug leakage from the lipid vesicles during storage and *in vivo* administration. The highest membrane permeability and fastest leakage of content is observed when bilayers are in the liquid-crystalline phase. Therefore, liposome stability can be enhanced by increasing the ratio of phospholipids with saturated and longer fatty acids chains, respectively, as well as addition of cholesterol, thus resulting in vesicles with rigid bilayers having higher transition temperature [127,129]. Furthermore, surface charge (both positive and negative) has also shown to affect liposome drug leakage in *in vivo* conditions [130]. This is a very unfavorable circumstance as the best antibacterial activities were achieved with positively charged or fluid (fusogenic) liposomes [58,81]. Moreover, the presence of anionic lipids in liposomal vesicles also favors binding to serum proteins to the vesicle surface [131]. Another aspect of the physical instability of liposomes is the aggregation and fusion upon storage leading to the increased vesicle size, subsequently affecting the *in vivo* performance.

Since the therapeutic outcome of liposomal antibiotics is determined by the achieved concentration of the drug at the site of action, high drug encapsulation efficiency is of great relevance. Encapsulation efficiency is closely associated with the concentration of the lipids building liposomes, applied preparation method and physicochemical properties of the encapsulated drug. It can be improved by optimization of the preparation process and raising the lipid concentration [35]. In this context, high doses of certain lipids can be potentially toxic, while some preparation method yielding high-encapsulation efficiency for hydrophilic drugs (e.g., reverse-evaporation method) are not suitable due to the possible presence of residual toxic organic solvents in the final liposomal formulations. As an alternative, freeze-dried liposome rehydration methods offering higher encapsulation efficiency compared to the conventional film hydration method, can be used.

Further important aspect of liposomal formulation is the ability for scaling up as the methods used on the laboratory scale are often complex and expensive. Large scale capacities are required for the preparation of marketed products providing sterile, well-characterized, and stable formulations. Unfortunately, the availability of certain production methods as well as the quality aspects depends on the characteristics of the lipids themselves. This limits the choice of liposome types from which one can select when optimizing liposome-based drug therapy. However, some of the methods have been used in industry, such as detergent removal method, the ethanol injection method and freeze-drying of bilayer-forming lipids in the presence of drug [132,133].

Finally, liposomal antibiotics aimed for parenteral delivery have to satisfy sterility requirement. Sterilization procedures for liposomal antibiotics generally cannot involve the use of heat, irradiation or chemical agents since lipids are very sensitive to high temperatures and easily undergo oxidation and hydrolysis reactions. Instead, the mechanical filtration is commonly used if the vesicles are sufficiently smaller than the bacterial cells. However, it still does not guarantee the removal of viral particles [41,132,133]. More innovative approaches in development of sterile liposome-based formulations are expected.

## 5. Perspectives

Bacterial biofilms are an undoubtedly challenging environment for drug delivery due to their physical composition, inter- and intra-species variations as well as the added complication of quorum sensing. Deeper insights on the mechanisms of biofilm survivals open up the possibilities to design novel agents able to combat the biofilms. All of the agents and drugs require a suitable carrier to improve therapeutic efficacy. A variety of liposomes, differing in the physicochemical features have been explored for improved delivery of antimicrobial agents to biofilm-forming microorganisms and mature biofilms [42]. Functionalizing liposomes with targeting ligands could be beneficial to achieve accumulation of the liposomes close to the bacterial cells and to promote close contact with bacteria. Targeting can be beneficial in environments with higher shear forces, such as oral cavity, where only short exposure times can be attained. The encapsulation of hydrophilic and lipophilic drugs in liposomes reduces their toxicity while improving the drugs’ effectiveness and increasing its therapeutic index [7]. It has been proven that liposomes increase intracellular drug concentrations through fusion with bacterial cell membrane [50]. In the case of CF, liposome encapsulation of antibiotics allows higher drug concentrations to be specifically delivered to the lungs, thereby improving aminoglycoside action against highly resistant pathogens [78].

Triggered drug release inside biofilms represents an attractive strategy to increase the local concentration of antimicrobial agent in the biofilm. Such approach has been utilized in the development of Arikace™, for triggered release of amikacin from DPPC/Chol liposomes at the site of infection (in the biofilm) [7,65]. Particularly interesting approaches to combat biofilms and microbial resistance involve the use of membrane building materials acting antimicrobially on their own, such as cationic lipids, metals and polymers. For instance, co-encapsulation of bismuth has shown to enhance antimicrobial activity of antibiotic loaded liposomes. Moreover, when bactericidal sub-inhibitory bismuth is combined with the solubilizing agent ethanedithiol, the decrease in antibiotic resistance through the inhibition of alginate production, bacterial respiratory enzymes and suppression of biofilm formation can be achieved [113,114].

Amongst the different novel strategies proposed for biofilm targeting the use of chitosan, a natural non-toxic biopolymer derived by deacetylation of chitin, has attracted considerable attention due to its ability to interfere with microbial virulence factors highly associated with formation of bacterial [120] and fungal [134] biofilms. In general, chitosan has shown stronger bactericidal effects against Gram-positive bacteria than Gram-negative bacteria, while the activity is inversely affected by pH [125]. Interestingly, even plain chitosan gel (medium *M*w) was capable to disrupt bacterial *P. aeruginosa* biofilms at concentrations as low as 0.13% at pH 4 [120]. Although chitosan alone might be potentially toxic for the cells, this limitation could be overcome by development of nanosystems additionally containing physiologically acceptable phospholipids. Therefore, nanosystems with increased antimicrobial effect might be achieved by combining liposomes-encapsulating antibiotics and chitosan, either as liposome coating material or gel-like vehicle for increasing viscosity of liposome dispersions.

## 6. Conclusions

Different strategies have been utilized to develop liposomes exhibiting improved anti-biofilm activity. The design of an optimal liposome formulation with desired anti-biofilm properties requires an optimal interplay of several factors including the biofilm characteristics, physicochemical properties of liposomes and the features of the encapsulated antimicrobial agent. Liposomes have shown promising results in the delivery of antibiotics in the therapy of chronic, biofilm-related infections due to their ability to lower the MICs and minimum biofilm inhibitory concentrations, and decrease the virulence factors in comparison to the conventional therapy. The clinical investigations on the formulations such as liposomal amikacin, ciprofloxacin and tobramycin have confirmed a reputable position of liposomal formulations among various innovative antimicrobial delivery systems. More work is required to assure optimal delivery of novel anti-biofilm agents designed and developed as a response to increased understanding of biofilm features.

## Figures and Tables

**Table 1:** Summary of the different types of liposomes investigated for delivery of antimicrobials to bacterial biofilms.

Liposome Type	Lipid Composition (Molar Ratio)	Drug	Average Size (nm)	Biofilm	Findings	Reference
**Conventional liposomes**	DPPC/Chol/SA (1:0.49:0.4)	-	126	*S. epidermidis*	Strong affinity for biofilm; lipid concentration and ionic strength of medium influenced the liposome adsorption to the biofilm	[59,60]
	DPPC/Chol (1:0.21) DPPC/Chol/DDAB (1:0.28:0.22)	Vancomycin, gentamicin	~120		Pronounced biofilm-inhibition effect with cationic liposomes	[61]
	DPPC/Chol/SA (variable mol% SA) DPPC/DPPG (variable mol% DPPG) DPPC/PI (variable mol% PI) DPPC/Chol/DDAB (11 mol% DDAB) DPPC/DC-Chol (32.3 mol% DC-Chol)	Triclosan, chlorhexidine	~100	*S. epidermidis P. vulgaris S. mutans S. sanguis*	Anionic and cationic liposomes superior in bactericide delivery as compared with the free bactericides; diffusion mechanism responsible for the bactericide delivery	[63]
	DPPC/Chol/SA (1:0.49:0.20–1.01) DPPC/DC-Chol (1:0.49:0.11–0.65) DPPC/Chol/DDAB (1:0.49:0.17–0.52)	Vancomycin	~120	*S. aureus*	Strong adsorption to the biofilm related to concentration of cationic lipid; DDAB-based liposomes inhibited bacterial growth more effectively than the free drug	[62]
	DMPC/Chol/DDAB (58.5:23:18.5) DMPC/PI (82.5:17.5) DPPC/Chol/SA (52.8:26:21.2)	Triclosan	108–129	*S. salivarius S. sanguis*	Linear relationship between liposome adsorption and mixed biofilm composition; anionic liposomes more effective than cationic	[51]
	DPPC/Chol/DC-Chol (1:0.49:0.43)	Benzyl penicillin G	~140	*S. aureus*	Effective delivery into biofilm at low drug concentrations and short exposure time	[64]
	DPPC/Chol (2:1)	Amikacin	~300	*P. aeruginosa*	Penetration into the biofilm and infected sputum; superior *in vivo* efficacy of inhaled liposomal amikacin in comparison to the free drug	[65]
	DMPC/Chol (2:1)	Tobramycin, gentamicin, amikacin	345–512		8–32-fold increased MBECs (non-mucoid strains) and 64–256-fold increased MBECs (mucoid strains), as compared to the corresponding MICs	[83]
	DPPC/Chol (6:1) DPPC/Chol/DCP (2:1:4) DPPC/Chol/DDAB (2:1:4)	Clarithro-mycin	170–220		Complete biofilm eradication with cationic and anionic liposomes; anionic liposomes superior (encapsulation efficiency, safety profile)	[71]
	DPPC/Chol (2:1) DOPC/DPPG (8:1)	Tobramycin	230–430	*B. cepacia*	No increased anti-biofilm activity relative to the free drug	[54]
	DPPC/Chol (6:1)	Azithromycin	406	*P. aeruginosa*	Significant reduction of bacterial growth in the biofilm and attenuation of virulence factors production	[72]
**Fusogenic liposomes**	DPPC/DMPG (15:1; 10:1)	Tobramycin	~400	*P. aeruginosa*	High bactericidal activity *in vivo* (rats)	[74]
	DPPC/DMPG (15:1; 18:1)		~400	*P. aeruginosa S. maltophilia B. cepacia S. aureus E. coli*	Strong activity at sub-MICs doses; increased penetration attained with dry powder preparations	[76,77]
	DPPC/DMPG (18:1)		~400	*P. aeruginosa*	Confirmed liposome fusion with bacterial cells	[50]
	DPPC/DMPG (10:1)		230–400	*B. cepacia*	Superior activity *in vivo* (rats); sustained drug release	[78]
	DOPE/DPPC/CHEMS (4:2:4)	Vancomycin	~100	*E. coli P. aeruginosa A. baumannii*	Enhanced penetration of the drug (adhesion and fusion mechanism)	[80]
	DPPC/DMPG liposomes (18:1) PC/DOPE/SA (1:1:0.7) PC/DOTAP/Chol (2.2:1:2.6)	Meropenem	107–142	*P. aeruginosa*	Fluidosomes showed higher MICs than the free drug did; increased activity of cationic liposomes (2–4-fold lower MICs than for the free drug)	[81]
	SPC/SCh (9.3:1)	Daptomycin	55	*S. aureus*	Powerful antimicrobial action *in vivo*; permeation into the skin; effective therapeutic concentrations maintained for several hours	[82]
**Surface-modified liposomes**	DMPC/DDAB/DPPE-PEG-2000 (80:20-11:0-9) DPPC/DDAB/DPPE-PEG-2000 (80:20-11:0-9) DSPC/DDAB/DPPE-PEG-2000 (80:20-11:0-9) DSPC/PI/DPPE-PEG-2000 (80:20-11:0-9)	-	~120	*S. aureus*	PEGylation reduced the liposomal anti-biofilm activity	[84]
	DPPC/SA/Chol/PEG (1:0.5:0.5:0.02) DPPC/DCP/Chol/PEG (1:0.5:0.5:0.02)	Vancomycin, rifampin	120–140	*S. epidermidis*	PEGylation did not reduce liposome anti-biofilm activity; increasing of incubation time enhanced the biofilm eradication effect	[85]
	DPPC/PI/DPPEMBS- anti- *oralis* antibody (82.8:2.6:14.6; 92.6:3.6:4.8) DPPC/Chol/SA/DPPEMBS- anti-*oralis* antibody (59:24.9:3.6:12.5)	-	~100	*S. oralis S. epidermidis*	Immunoliposomes showed greater antimicrobial affinity than the antibody-free liposomes	[86]
	DPPC/PI/DPPEMBS- anti- *oralis* antibody (1:0.03:0.14)	-	~80–120		Immunoliposomes adsorbed to the surface of *S. oralis*; cationic liposomes had greater affinity for *S. epidermidis* biofilm than anionic liposomes	[87]
	DPPC/PI/DPPEMBS-anti- *oralis* antibody (82.8:2.6:14.6; 92.6:3.6:4.8)	Triclosan chlorhexidine	~100		Greater antibacterial activity than with the free drug; the extent of growth inhibition linearly related to the number of liposomes targeted to biofilm surface	[55]
	PC/Chol/SA (2:1:0.1) (PC/Chol/SA)/ConA (0.1):1	Metronidazole	~3000	*S. mutans*	Targeting the surface “glyco-calyx” of biofilm; inhibition of bacterial growth	[56]
	PC/Chol/SA (7:2:1) (PC/Chol/SA)/CHM (5):1 (PC/Chol/SA)/SM (7):1		400–450	*S. aureus*	Mannosylated liposomes showed increased ability to target biofilms; superior targeting ability with SM-based liposomes	[88]
**Reactive liposomes**	DPPC/PI (4.85:1)	GO GO+HRP CPO+GO LPO+GO	97–224	*S. gordonii*	Inhibition increases with liposome-biofilm and substrate-biofilm incubation time and extent of enzyme encapsulation	[89,90]
**Metal co-encapsulating liposomes**	DPPC/DMPG (1:1)	Gentamicin-gallium	~300	*P. aeruginosa*	Eradication of the biofilm and interruption of QS signaling	[91]
	DSPC/Chol (2:1)	Tobramycin- BiEDT	~900	*P. aeruginosa*	Eradication of the biofilm at significantly lower concentrations than with the free BiEDT; less toxicity of the liposomal formulation; QS suppressing properties; deeper penetration into the biofilm; attenuation of the alginate production; reduction of bacterial counts in the lungs of infected rats *in vivo* but without complete eradication	[92,93,94,95,96]
**SSLs**	DPPC/PI (PI-14 mol%), DPPC/DDAB/Chol (DDAB-14 mol%) adsorbed on zinc citrate particles	Triclosan, penicillin G	~100	*S. oralis*	Activity affected by the amount of the lipid adsorbed onto zinc citrate particles; no particular advancement in comparison to individual constituents	[97]
	DPPC/Chol/SA (1:0.43:0.49), impregated in CS	Gentamicin	n.d.	*S. aureus*	Significantly increased efficacy as compared to liposomal gentamicin; complete sterilization of bone tissues; prolonged drug release	[98]
**LLSs**	DPPC/Chol (3:1), β-TCP	Gentamicin	~110–5200	*S. aureus*	Superior antibiofilm activity achieved with 800 nm-sized liposomes; controlled drug delivery	[99]
	SPC/SA/Chol (7:3:1), n-HA/CS/KGM	Vancomycin	~200		Successful inhibition of the biofilm formation; sustained release from LLS	[100]
	SPC/SA/Chol (7:1:1), β-TCP	Ceftazimide	~160		Significant *in vitro* anti-biofilm activity during the longer incubation time; sustained drug release	[101]
**BBLs**	DSPC/Chol/ALN-TEG-Chol	Oxacillin	~100	*S. aureus*	Significantly higher antibacterial effects than with DSPC/Chol liposomes; fast and strong binding to hydroxyapatite	[102]
**Liposomes-in-hydrogel**	SPC, chitosan hydrogel	Mupirocin	920	*S. aureus*	Prevention of biofilm formation; reduced cytotoxicity; *in vivo* efficacy comparable to the marketed product	[103]
	DPPC/Chol/PEG-DSPE/rhodamine-DPPE (1:1:0.05:0.001), PEG-gelatin hydrogel	Ciprofloxacin	~100	*P. aeruginosa*	Prevention of bacterial adhesion and biofilm formation on urinary catheters; prolonged drug release; improved biocompatibility of coated catheters; effective delay of the bateriuria *in vivo*	[104,105]

ALN-TEG-Chol, alendronate-tri(ethyleneglycol)-cholesterol conjugate; BBLs, biomineral-binding liposomes; β-TCP, β-tricalcium phosphate; BiEDT, bismuth-ethanedithiol; CHEMS, cholesterol hemisuccinate; CHM, cholesteryl mannan; Chol, cholesterol; ConA, concanavalin A; CPO, chloroperoxidase; CS, calcium sulfate; DC-Chol, dimethylammonium ethane carbamoyl cholesterol; DCP, dicetylphosphate; DDAB: dimethyldioctadecylammonium bromide; DMPC; dimyristoylphosphatidylcholine; DMPG, dimyristoylphosphatidylglycerol; DOPC, dioleoylphophatidylcholine; DOPE, dioleoylphosphatidylethanolamine; DOTAP, 1,2-oleoyl-3-trimethylammonium-propan; DPPC, dipalmitoylphosphatidylcholine; DPPE, diplalmitoylphosphatidylethanolamine; DPPEMBS, maleimidobenzoyl-*N*-hydroxysuccinimide(MBS) derivative of DPPE; DPPG, dipalmitoylphosphatidylglycerol; DSPC, distearoylphosphatidylcholine; GO, glucose oxidase; GO-HRP, glucose oxidase-horse radish peroxidase; LLSs, liposome loaded scaffolds; LPO, lactoperoxidase; MBEC, minimal biofilm eradication concentration; MIC, minimal inhibitory concentration; n.d., not determined; n-HA/CS/KGM, nano-hydroxyapatite/chitosan/konjac glucomannan; PC, phosphatidylcholine; PEG, poly(ethylene) glycol; PI, phosphatidylinositol; QS, quorum sensing; SA, stearylamine; SCh, sodium cholate; SM, sialo-mannan; SSLs, solid supported liposomes; SPC, soy phosphatidylcholine (lecithin).

## Abbreviations

The following abbreviations are used in this manuscript:

ALN-TEG-Chol	alendronate-tri(ethyleneglycol)-cholesterol conjugate
BBLs	biomineral-binding liposomes
βTCP	β-tricalcium phosphate
BiEDT	bismuth-ethanedithiol
CF	cystic fibrosis
CHM	cholesteryl mannan
Chol	cholesterol
CHEMS	cholesteryl hemisuccinate
ConA	concanavalin A
CPO	chloroperoxidase
CS	calcium sulfate
CS/KGM	chitosan/konjac glucomannan
DC-Chol	dimethylammonium ethane carbamoyl cholesterol
DCP	dicetylphosphate
DDAB	dimethyldioctadecylammonium bromide
DMPC	dimyristoylphosphatidylcholine
DMPG	dimyristoylphosphatidylglycerol
DOPC	dioleoylphophatidylcholine
DOPE	dioleoylphosphatidylethanolamine
DOTAP	1,2-oleoyloxy-3-trimethylammonium-propan
DPPC	dipalmitoylphosphatidylcholine
DPPE	diplalmitoylphosphatidylethanolamine
DPPEMBS	maleimidobenzoyl-*N*-hydroxysuccinimide (MBS) derivative of DPPE
DPPG	dipalmitoylphosphatidylglycerol
DSPC	distearoylphosphatidylcholine
GO	glucose oxidase
GO-HRP	glucose oxidase-horse radish peroxidase
HSPC	hydrogenated soy phosphatidylcholine
LipoBiEDT-TOB	liposomal BiEDT-loaded tobramycin
Lipo-Ga-GEN	liposomal formulation with gentamicin and gallium
LLS	liposome loaded scaffold
LPO	lactoperoxidase
MBC	minimal bactericidal concentration
MBEC	minimal biofilm eradication concentration
MIC	minimal inhibitory concentration
n-HA/CS/KGM	nano-hydroxyapatite/chitosan/konjac glucomannan
PC	phosphatidylcholine
PEG	poly(ethylene) glycol
PI	phosphatidylinositol
QS	quorum sensing
SA	stearylamine
SCh	sodium cholate
SM	sialo-mannan
SPC	soy phosphatidylcholine (lecithin)
SSLs	solid supported liposomes

## References

[1] Flemming H.C., Wingender J. (2010). The biofilm matrix. Nat. Rev. Microbiol..

[2] Hall-Stoodley L., Costerton J.W., Stoodley P. (2004). Bacterial biofilms: From the natural environment to infectious diseases. Nat. Rev. Microbiol..

[3] Greenberg E.P. (2003). Bacterial communication and group behavior. J. Clin. Investig..

[4] Hentzer M., Givskov M. (2003). Pharmacological inhibition of quorum sensing for the treatment of chronic bacterial infections. J. Clin. Investig..

[5] Hoiby N., Bjarnsholt T., Givskov M., Molin S., Ciofu O. (2010). Antibiotic resistance of bacterial biofilms. Int. J. Antimicrob. Agents.

[6] Stoodley P., Sauer K., Davies D.G., Costerton J.W. (2002). Biofilms as complex differentiated communities. Annu. Rev. Microbiol..

[7] Forier K., Raemdonck K., De Smedt S.C., Demeester J., Coenye T., Braeckmans K. (2014). Lipid and polymer nanoparticles for drug delivery to bacterial biofilms. J. Control. Release.

[8] Nafee N., Rai M., Kon K. (2015). Nanocarriers against bacterial biofilms: Current status and future perspectives. Nanotechnology in Diagnosis, Treatment and Prophylaxis of Infectious Diseases.

[9] Nickel J.C., McLean R.J.C. (1998). Bacterial biofilms in urology. Infect. Urol..

[10] Percival S.L., Bowler P.G. (2004). Biofilms and. their potential role in wound healing. Wounds.

[11] Scheie A.A., Petersen F.C. (2004). The biofilm concept: Consequences for future prophylaxis of oral diseases?. Crit. Rev. Oral Biol. Med..

[12] Singh P.K., Schaefer A.L., Parsek M.R., Moninger T.O., Welsh M.J., Greenberg E.P. (2000). Quorum-sensing signals indicate that cystic fibrosis lungs are infected with bacterial biofilms. Nature.

[13] Post J.C. (2001). Direct evidence of bacterial biofilms in otitis media. Laryngoscope.

[14] Sanderson A.R., Leid J.G., Hunsaker D. (2006). Bacterial Biofilms on the sinus mucosa of human subjects with chronic rhinosinusitis. Laryngoscope.

[15] Boumis E., Gesu G., Menichetti F., Ranieri M., Rinaldi M., Suter F., Nicastri E., Lauria F.N., Carosi G., Moroni M. (2010). Consensus document on controversial issues in the diagnosis and treatment of bloodstream infections and endocarditis. Int. J. Infect. Dis..

[16] Costerton W., Veeh R., Shirtliff M., Pasmore M., Post C., Ehrlich G. (2003). The application of biofilm science to the study and control of chronic bacterial infections. J. Clin. Investig..

[17] Mah T.F.C., O’Toole G.A. (2001). Mechanisms of biofilm resistance to antimicrobial agents. Trends Microbiol..

[18] Smith A.W. (2005). Biofilms and antibiotic therapy: Is there a role for combating bacterial resistance by the use of novel drug delivery systems?. Adv. Drug Deliver. Rev..

[19] Sihorkar V., Vyas S.P. (2001). Biofilm consortia on biomedical and biological surfaces: Delivery and targeting strategies. Pharm. Res..

[20] Sousa C., Bothello C., Oliveria R., Medez-Vilas A. (2011). Nanotechnology applied to medical biofilms control. Science against Microbial Pathogens: Communicating Current Research and Technological Advances.

[21] Sun F.J., Qu F., Ling Y., Mao P.Y., Xia P.Y., Chen H.P., Zhou D.S. (2013). Biofilm-associated infections: Antibiotic resistance and novel therapeutic strategies. Future Microbiol..

[22] Stewart P.S., Franklin M.J. (2008). Physiological heterogeneity in biofilms. Nat. Rev. Microbiol..

[23] Donlan R.M., Costerton J.W. (2001). Biofilms: Survival mechanisms of clinically relevant microorganisms. Clin. Microbiol. Rev..

[24] Donelli G., Francolini I., Piozzi A., Di Rosa R., Marconi W. (2002). New polymer-antibiotic systems to inhibit bacterial biofilm formation: A suitable approach to prevent central venous catheter-associated infections. J. Chemother..

[25] Piozzi A., Francolini I., Occhiaperti L., Venditti M., Marconi W. (2004). Antimicrobial activity of polyurethanes coated with antibiotics: A new approach to the realization of medical devices exempt from microbial colonization. Int. J. Pharm..

[26] Breuing K.H., Bayer L., Neuwalder J., Orgill D.P. (2005). Early experience using low-frequency ultrasound in chronic wounds. Ann. Plast. Surg..

[27] Stanisic M.M., Provo B.J., Larson D.L., Kloth L.C. (2005). Wound debridement with 25 kHz ultrasound. Adv. Skin Wound Care.

[28] Wolcott R.D., Rumbaugh K.P., James G., Schultz G., Phillips P., Yang Q., Watters C., Stewart P.S., Dowd S.E. (2010). Biofilm maturity studies indicate sharp debridement opens a time-dependent therapeutic window. J. Wound Care.

[29] Rasmussen T.B., Givskov M. (2006). Quorum-sensing inhibitors as anti-pathogenic drugs. Int. J. Med. Microbiol..

[30] Matl F.D., Obermeier A., Zlotnyk J., Friess W., Stemberger A., Burgkart R. (2011). Augmentation of antibiotic activity by low-frequency electric and electromagnetic fields examining *Staphylococcus aureus* in broth media. Bioelectromagnetics.

[31] Ensing G.T., Roeder B.L., Nelson J.L., van Horn J.R., van der Mei H.C., Busscher H.J., Pitt W.G. (2005). Effect of pulsed ultrasound in combination with gentamicin on bacterial viability in biofilms on bone cements *in vivo*. J. Appl. Microbiol..

[32] Soukos N.S., Socransky S.S., Mulholland S.E., Lee S., Doukas A.G. (2000). Photomechanical drug delivery into bacterial biofilms. Pharm. Res..

[33] Kasimanickam R.K., Ranjan A., Asokan G.V., Kasimanickam V.R., Kastelic J.P. (2013). Prevention and treatment of biofilms by hybrid- and nanotechnologies. Int. J. Nanomed..

[34] Tamilvanan S., Venkateshan N., Ludwig A. (2008). The potential of lipid- and polymer-based drug delivery carriers for eradicating biofilm consortia on device-related nosocomial infections. J. Control. Release.

[35] Drulis-Kawa Z., Dorotkiewicz-Jach A. (2010). Liposomes as delivery systems for antibiotics. Int. J. Pharm..

[36] Alhariri M., Azghani A., Omri A. (2013). Liposomal antibiotics for the treatment of infectious diseases. Expert Opin. Drug Deliv..

[37] Basnet P., Škalko-Basnet N. (2013). Nanodelivery systems for improved topical antimicrobial therapy. Curr. Pharm. Des..

[38] Fenske D.B., Cullis P.R. (2008). Liposomal nanomedicines. Expert Opin. Drug Deliv..

[39] Torchilin V.P. (2005). Recent advances with liposomes as pharmaceutical carriers. Nat. Rev. Drug Discov..

[40] Vanić Ž., Holaeter A.M., Škalko-Basnet N. (2015). (Phospho)lipid-based nanosystems for skin administration. Curr. Pharm. Des..

[41] Abed N., Couvreur P. (2014). Nanocarriers for antibiotics: A promising solution to treat intracellular bacterial infections. Int. J. Antimicrob. Agents.

[42] Martin C., Low W.L., Gupta A., Amin M.C., Radecka I., Britland S.T., Raj P., Kenward K.M. (2015). Strategies for antimicrobial drug delivery to biofilm. Curr. Pharm. Des..

[43] Zhang L., Pornpattananangku D., Hu C.M., Huang C.M. (2010). Development of nanoparticles for antimicrobial drug delivery. Curr. Med. Chem..

[44] Aljuffali I.A., Huang C.H., Fang J.Y. (2015). Nanomedical strategies for targeting skin microbiomes. Curr. Drug Metab..

[45] Vanić Ž., Hurler J., Ferderber K., Golja Gašparović P., Škalko-Basnet N., Filipović-Grčić J. (2014). Novel vaginal drug delivery system: Deformable propylene glycol liposomes-in-hydrogel. J. Liposome Res..

[46] Vanić Ž., Škalko-Basnet N. (2013). Nanopharmaceuticals for improved topical vaginal therapy: Can they deliver?. Eur. J. Pharm. Sci..

[47] Cipolla D., Shekunov B., Blanchard J., Hickey A. (2014). Lipid-based carriers for pulmonary products: Preclinical development and case studies in humans. Adv. Drug Deliv. Rev..

[48] Hadinoto K., Cheow W.S. (2014). Nano-antibiotics in chronic lung infection therapy against *Pseudomonas aeruginosa*. Colloids Surf. B.

[49] Mishra G.P., Bagui M., Tamboli V., Mitra A.K. (2011). Recent applications of liposomes in ophthalmic drug delivery. J. Drug Deliv..

[50] Sachetelli S., Khalil H., Chen T., Beaulac C., Senechal S., Lagace J. (2000). Demonstration of a fusion mechanism between a fluid bactericidal liposomal formulation and bacterial cells. BBA-Biomembranes.

[51] Robinson A.M., Bannister M., Creeth J.E., Jones M.N. (2001). The interaction of phospholipid liposomes with mixed bacterial biofilms and their use in the delivery of bactericide. Colloid. Surface A.

[52] Drulis-Kawa Z., Dorotkiewicz-Jach A., Gubernator J., Gula G., Bocer T., Doroszkiewicz W. (2009). The interaction between *Pseudomonas aeruginosa* cells and cationic PC:Chol:DOTAP liposomal vesicles *versus* outer-membrane structure and envelope properties of bacterial cell. Int. J. Pharm..

[53] Dong D., Thomas N., Thierry B., Vreugde S., Clive A., Prestidge C.A., Wormald P.-J. (2015). Distribution and inhibition of liposomes on *Staphylococcus aureus* and *Pseudomonas aeruginosa* biofilm. PLoS ONE.

[54] Messiaen A.S., Forier K., Nelis H., Braeckmans K., Coenye T. (2013). Transport of nanoparticles and tobramycin-loaded liposomes in *Burkholderia cepacia* complex biofilms. PLoS ONE.

[55] Robinson A.M., Creeth J.E., Jones M.N. (2000). The use of immunoliposomes for specific delivery of antimicrobial agents to oral bacteria immobilized on polystyrene. J. Biomat. Sci. Polym. Ed..

[56] Vyas S.P., Sihorkar V., Dubey P.K. (2001). Preparation, characterization and *in vitro* antimicrobial activity of metronidazole bearing lectinized liposomes for intra-periodontal pocket delivery. Pharmazie.

[57] Strathmann M., Wingender J., Flemming H.C. (2002). Application of fluorescently labelled lectins for the visualization and biochemical characterization of polysaccharides in biofilms of *Pseudomonas aeruginosa*. J. Microbiol. Methods.

[58] Gubernator J., Drulis-Kawa Z., Dorotkiewicz-Jach A., Doroszkiewicz W., Kozubek A. (2007). *In vitro* antimicrobial activity of liposomes containing ciprofloxacin, meropenem and gentamicin against gram-negative clinical bacterial strains. Lett. Drug Des. Discov..

[59] Sanderson N.M., Jones M.N. (1996). Targeting of cationic liposomes to skin-associated bacteria. Pestic. Sci..

[60] Sanderson N.M., Guo B.Q., Jacob A.E., Handley P.S., Cunniffe J.G., Jones M.N. (1996). The interaction of cationic liposomes with the skin-associated bacterium Staphylococcus epidermidis: Effects of ionic strength and temperature. BBA-Biomembranes.

[61] Sanderson N.M., Jones M.N. (1996). Encapsulation of vancomycin and gentamicin within cationic liposomes for inhibition of growth of *Staphylococcus epidermidis*. J. Drug Target..

[62] Kim H.J., Gias E.L.M., Jones M.N. (1999). The adsorption of cationic liposomes to *Staphylococcus aureus* biofilms. Colloid. Surface A.

[63] Jones M.N., Song Y.H., Kaszuba M., Reboiras M.D. (1997). The interaction of phospholipid liposomes with bacteria and their use in the delivery of bactericides. J. Drug Target..

[64] Kim H.J., Jones M.N. (2004). The delivery of benzyl penicillin to *Staphylococcus aureus* biofilms by use of liposomes. J. Liposome Res..

[65] Meers P., Neville M., Malinin V., Scotto A.W., Sardaryan G., Kurumunda R., Mackinson C., James G., Fisher S., Perkins W.R. (2008). Biofilm penetration, triggered release and *in vivo* activity of inhaled liposomal amikacin in chronic *Pseudomonas aeruginosa* lung infections. J. Antimicrob. Chemother..

[66] Clancy J.P., Dupont L., Konstan M.W., Billings J., Fustik S., Goss C.H., Lymp J., Minic P., Quittner A.L., Rubenstein R.C. (2013). Phase II studies of nebulised Arikace in CF patients with *Pseudomonas aeruginosa* infection. Thorax.

[67] Bilton D., Pressler T., Fajac I., Clancy J.P., Sands D., Minic P., Cipolli M., LaRosa M., Galeva I., Amparo Sole A. Phase 3 efficacy and safety data from randomized, multicenter study of liposomal amikacin for inhalation (Arikace^®^) compared with TOBI^®^ in cystic fibrosis patients with chronic infection due to *Pseudomonas aeruginosa*. Proceedings of the North American Cystic Fibrosis Conference (NACFC).

[68] Waters V., Ratjen F. (2014). Inhaled liposomal amikacin. Expert Rev. Respir. Med..

[69] Bruinenberg P., Blanchard J.D., Cipolla D., Dayton F., Mudumga S., Gonda I., Byron P.R., Dalby R.N., Peart J., Suman J.D., Farr S.J., Young P.M. (2010). Inhaled liposomal ciprofloxacin: Once a day management of respiratory infections. Respiratory Drug Delivery.

[70] Cipolla D., Blanchard J., Gonda I. (2016). Development of liposomal ciprofloxacin to treat lung infections. Pharmaceutics.

[71] Alhajlan M., Alhariri M., Omri A. (2013). Efficacy and safety of liposomal clarithromycin and its effect on *Pseudomonas aeruginosa* virulence factors. Antimicrob. Agents Chemother..

[72] Solleti V.S., Alhariri M., Halwani M., Omri A. (2015). Antimicrobial properties of liposomal azithromycin for Pseudomonas infections in cystic fibrosis patients. J. Antimicrob. Chemoth..

[73] Cevc G. (1991). How membrane chain-melting phase-transition temperature is affected by the lipid chain asymmetry and degree of unsaturation: An effective chain-length model. Biochemistry.

[74] Beaulac C., Clement-Major S., Hawari J., Lagace J. (1996). Eradication of mucoid *Pseudomonas aeruginosa* with fluid liposome-encapsulated tobramycin in an animal model of chronic pulmonary infection. Antimicrob. Agents Chemother..

[75] Sachetelli S., Beaulac C., Riffon R., Lagace J. (1999). Evaluation of the pulmonary and systemic immunogenicity of Fluidosomes, a fluid liposomal-tobramycin formulation for the treatment of chronic infections in lungs. BBA-Gen. Subjects.

[76] Beaulac C., Sachetelli S., Lagace J. (1998). *In-vitro* bactericidal efficacy of sub-MIC concentrations of liposome-encapsulated antibiotic against gram-negative and gram-positive bacteria. J. Antimicrob. Chemother..

[77] Beaulac C., Sachetelli S., Lagace J. (1993). Aerosolization of low phase transition temperature liposomal tobramycin as a dry powder in an animal model of chronic pulmonary infection caused by *Pseudomonas aeruginosa*. J. Drug Target..

[78] Marier J.F., Lavigne J., Ducharme M.P. (2002). Pharmacokinetics and efficacies of liposomal and conventional formulations of tobramycin after intratracheal administration in rats with pulmonary *Burkholderia cepacia* infection. Antimicrob. Agents Chemother..

[79] Inhaled Liposomal Tobramycin—Axentis Pharma AG. http://adisinsight.springer.com/drugs/800025294.

[80] Nicolosi D., Scalia M., Nicolosi V.M., Pignatello R. (2010). Encapsulation in fusogenic liposomes broadens the spectrum of action of vancomycin against Gram-negative bacteria. Int. J. Antimicrob. Agents.

[81] Drulis-Kawa Z., Gubernator J., Dorotkiewicz-Jach A., Doroszkiewicz W., Kozubek A. (2006). *In vitro* antimicrobial activity of liposomal meropenem against *Pseudomonas aeruginosa* strains. Int. J. Pharm..

[82] Li C., Zhang X., Huang X., Wang X., Liao G., Chen Z. (2013). Preparation and characterization of flexible nanoliposomes loaded with daptomycin, a novel antibiotic, for topical skin therapy. Int. J. Nanomed..

[83] Alipour M., Suntres Z.E., Omri A. (2009). Importance of DNase and alginate lyase for enhancing free and liposome encapsulated aminoglycoside activity against *Pseudomonas aeruginosa*. J. Antimicrob. Chemother..

[84] Ahmed K., Muiruri P.W., Jones G.H., Scott M.J., Jones M.N. (2001). The effect of grafted poly(ethylene glycol) on the electrophoretic properties of phospholipid liposomes and their adsorption to bacterial biofilms. Colloids Surface A.

[85] Moghadas-Sharif N., Fazly Bazzaz B.S., Khameneh B., Malaekeh-Nikouei B. (2015). The effect of nanoliposomal formulations on Staphylococcus epidermidis biofilm. Drug Dev. Ind. Pharm..

[86] Robinson A.M., Creeth J.E., Jones M.N. (1998). The specificity and affinity of immunoliposome targeting to oral bacteria. BBA-Biomembranes.

[87] Kaszuba M., Robinson A.M., Song Y.H., Creeth J.E., Jones M.N. (1997). The visualisation of the targeting of phospholipid liposomes to bacteria. Colloids Surface B.

[88] Vyas S.P., Sihorkar V., Jain S. (2007). Mannosylated liposomes for bio-film targeting. Int. J. Pharm..

[89] Hill K.J., Kaszuba M., Creeth J.E., Jones M.N. (1997). Reactive liposomes encapsulating a glucose oxidase-peroxidase system with antibacterial activity. BBA-Biomembranes.

[90] Jones M.N., Hill K.J., Kaszuba M., Creeth J.E. (1998). Antibacterial reactive liposomes encapsulating coupled enzyme systems. Int. J. Pharm..

[91] Halwani M., Yebio B., Suntres Z.E., Alipour M., Azghani A.O., Omri A. (2008). Co-encapsulation of gallium with gentamicin in liposomes enhances antimicrobial activity of gentamicin against *Pseudomonas aeruginosa*. J. Antimicrob. Chemother..

[92] Halwani M., Blomme S., Suntres Z.E., Alipour M., Azghani A.O., Kumar A., Omri A. (2008). Liposomal bismuth-ethanedithiol formulation enhances antimicrobial activity of tobramycin. Int. J. Pharm..

[93] Halwani M., Hebert S., Suntres Z.E., Lafrenie R.M., Azghani A.O., Omri A. (2009). Bismuth-thiol incorporation enhances biological activities of liposomal tobramycin against bacterial biofilm and quorum sensing molecules production by *Pseudomonas aeruginosa*. Int. J. Pharm..

[94] Alipour M., Dorval C., Suntres Z.E., Omri A. (2011). Bismuth-ethanedithiol incorporated in a liposome-loaded tobramycin formulation modulates the alginate levels in mucoid *Pseudomonas aeruginosa*. J. Pharm. Pharmacol..

[95] Alipour M., Suntres Z.E., Lafrenie R.M., Omri A. (2010). Attenuation of *Pseudomonas aeruginosa* virulence factors and biofilms by co-encapsulation of bismuth-ethanedithiol with tobramycin in liposomes. J. Antimicrob. Chemother..

[96] Alhariri M., Omri A. (2013). Efficacy of liposomal bismuth-ethanedithiol-loaded tobramycin after intratracheal administration in rats with pulmonary *Pseudomonas aeruginosa* infection. Antimicrob. Agents Chemother..

[97] Catuogno C., Jones M.N. (2003). The antibacterial properties of solid supported liposomes on Streptococcus oralis biofilms. Int. J. Pharm..

[98] Tang H., Xu Y.Q., Zheng T., Li G., You Y.G., Jiang M.Y., Li J., Ding J. (2009). Treatment of osteomyelitis by liposomal gentamicin-impregnated calcium sulfate. Arch. Orthop. Traum. Surg..

[99] Zhu C.T., Xu Y.Q., Shi J.A., Li J., Ding J. (2010). Liposome combined porous beta-TCP scaffold: Preparation, characterization, and anti-biofilm activity. Drug Deliv..

[100] Ma T., Shang B.C., Tang H., Zhou T.H., Xu G.L., Li H.L., Chen Q.H., Xu Y.Q. (2011). Nano-hydroxyapatite/chitosan/konjac glucomannan scaffolds loaded with cationic liposomal vancomycin: Preparation, *in vitro* release and activity against *Staphylococcus aureus* biofilms. J. Biomat. Sci. Polym. Ed..

[101] Zhou T.H., Su M., Shang B.C., Ma T., Xu G.L., Li H.L., Chen Q.H., Sun W., Xu Y.Q. (2012). Nano-hydroxyapatite/beta-tricalcium phosphate ceramics scaffolds loaded with cationic liposomal ceftazidime: preparation, release characteristics *in vitro* and inhibition to *Staphylococcus aureus* biofilms. Drug Dev. Ind. Pharm..

[102] Liu X.M., Zhang Y.J., Chen F., Khutsishvili I., Fehringer E.V., Marky L.A., Bayles K.W., Wang D. (2012). Prevention of orthopedic device-associated osteomyelitis using oxacillin-containing biomineral-binding liposomes. Pharm. Res..

[103] Hurler J., Sorensen K.K., Fallarero A., Vuorela P., Škalko-Basnet N. (2013). Liposomes-in-hydrogel delivery system with mupirocin: *In vitro* antibiofilm studies and *in vivo* evaluation in mice burn model. Biomed. Res. Int..

[104] DiTizio V., Ferguson G.W., Mittelman M.W., Khoury A.E., Bruce A.W., DiCosmo F. (1998). A liposomal hydrogel for the prevention of bacterial adhesion to catheters. Biomaterials.

[105] Pugach J.L., DiTizio V., Mittelman M.W., Bruce A.W., DiCosmo F., Khoury A.E. (1999). Antibiotic hydrogel coated Foley catheters for prevention of urinary tract infection in a rabbit model. J. Urol..

[106] Kaszuba M., Jones M.N. (1999). Hydrogen peroxide production from reactive liposomes encapsulating enzymes. BBA-Biomembranes.

[107] Olakanmi O., Britigan B.E., Schlesinger L.S. (2000). Gallium disrupts iron metabolism of mycobacteria residing within human macrophages. Infect. Immun..

[108] Harrington J.R., Martens R.J., Cohen N.D., Bernstein L.R. (2006). Antimicrobial activity of gallium against virulent *Rhodococcus equiin vitro* and *in vivo*. J. Vet. Pharmacol. Ther..

[109] Kaneko Y., Thoendel M., Olakanmi O., Britigan B.E., Singh P.K. (2007). The transition metal gallium disrupts *Pseudomonas aeruginosa* iron metabolism and has antimicrobial and antibiofilm activity. J. Clin. Investig..

[110] Antunes L.C.S., Imperi F., Minandri F., Visca P. (2012). *In Vitro* and *in vivo* antimicrobial activities of gallium nitrate against multidrug-resistant *Acinetobacter baumannii*. Antimicrob. Agents Chemother..

[111] Domenico P., Baldassarri L., Schoch P.E., Kaehler K., Sasatsu M., Cunha B.A. (2001). Activities of bismuth thiols against staphylococci and staphylococcal biofilms. Antimicrob. Agents Chemother..

[112] Domenico P., Reich J., Madonia W., Cunha B.A. (1996). Resistance to bismuth among gram-negative bacteria is dependent upon iron and its uptake. J. Antimicrob. Chemother..

[113] Veloira W.G., Domenico P., LiPuma J.J., Davis J.M., Gurzenda E., Kazzaz J.A. (2003). *In vitro* activity and synergy of bismuth thiols and tobramycin against *Burkholderia cepacia* complex. J. Antimicrob. Chemother..

[114] Huang C.T., Stewart P.S. (1999). Reduction of polysaccharide production in *Pseudomonas aeruginosa* biofilms by bismuth dimercaprol (BisBAL) treatment. J. Antimicrob. Chemother..

[115] Folsom J.P., Baker B., Stewart P.S. (2011). *In vitro* efficacy of bismuth thiols against biofilms formed by bacteria isolated from human chronic wounds. J. Appl. Microbiol..

[116] Varposhti M., Abdi Ali A., Mohammadi P. (2014). Synergistic effects of bismuth thiols and barious antibiotics against *Pseudomonas aeruginosa* biofilm. Jundishapur J. Microbiol..

[117] Hurler J., Berg O.A., Skar M., Conradi A.H., Johnsen P.J., Škalko-Basnet N. (2012). Improved burns therapy: Liposomes-in-hydrogel delivery system for mupirocin. J. Pharm. Sci..

[118] Palac Z., Hurler J., Škalko-Basnet N., Filipović-Grčić J., Vanić Ž. (2015). Elastic liposomes-in-vehicle formulations destined for skin therapy: The synergy between type of liposomes and vehicle. Drug Dev. Ind. Pharm..

[119] Pavelić Ž., Škalko-Basnet N., Filipović-Grčić J., Martinac A., Jalšenjak I. (2005). Development and *in vitro* evaluation of a liposomal vaginal delivery system for acyclovir. J. Control. Release.

[120] Kandimalla K.K., Borden E., Omtri R.S., Boyapati S.P., Smith M., Lebby K., Mulpuru M., Gadde M. (2013). Ability of chitosan gels to disrupt bacterial biofilms and their applications in the treatment of bacterial vaginosis. J. Pharm. Sci..

[121] Ribeiro M.P., Espiga A., Silva D., Baptista P., Henriques J., Ferreira C., Silva J.C., Borges J.P., Pires E., Chaves P. (2009). Development of a new chitosan hydrogel for wound dressing. Wound Repair Regen..

[122] Bradshaw D.J., Marsh P.D., Watson G.K., Cummins D. (1993). The effects of triclosan and zinc citrate, alone and in combination, on a community of oral bacteria grown-*in vitro*. J. Dent. Res..

[123] Zhou G., Li Y., Zhang L., Zuo Y., Jansen J.A. (2007). Preparation and characterization of nano-hydroxyapatite/chitosan/konjac glucomannan composite. J. Biomed. Mater. Res. A.

[124] Zheng L.Y., Zhu J.A.F. (2003). Study on antimicrobial activity of chitosan with different molecular weights. Carbohyd. Polym..

[125] No H.K., Park N.Y., Lee S.H., Meyers S.P. (2002). Antibacterial activity of chitosans and chitosan oligomers with different molecular weights. Int. J. Food Microbiol..

[126] Yamakami K., Tsumori H., Sakurai Y., Shimizu Y., Nagatoshi K., Sonomoto K. (2013). Sustainable inhibition efficacy of liposome-encapsulated nisin on insoluble glucan-biofilm synthesis by *Streptococcus mutans*. Pharm. Biol..

[127] New R.R.C. (1990). Liposomes: A Practical Approach.

[128] Škalko-Basnet N., Vanić Ž., Boukherroub R., Szunerits S., Drider D. Lipid-based nanopharmaceuticals in antimicrobial therapy. Functionalized Nanomaterials for the Management of Microbial Infection.

[129] Kirby C., Gregoriadis G., Mathiowith E. (1999). Liposomes. Encyclopedia of Controlled Drug Delivery.

[130] Ulrich A.S. (2002). Biophysical aspects of using liposomes as delivery vehicles. Biosci. Rep..

[131] Briones E., Colino C.I., Lanao J.M. (2008). Delivery systems to increase the selectivity of antibiotics in phagocytic cells. J. Control. Release.

[132] Wagner A., Vorauer-Uhl K. (2011). Liposome technology for industrial purposes. J. Drug Deliv..

[133] Storm G., Crommelin D.J.A. (1998). Liposomes: Quo vadis?. Pharm. Sci. Technol. Today.

[134] Silva-Dias A., Palmeira-De-Oliveira A., Miranda I.M., Branco J., Cobrado L., Monteiro-Soares M., Queiroz J.A., Pina-Vaz C., Rodrigues A.G. (2014). Anti-biofilm activity of low-molecular weight chitosan hydrogel against Candida species. Med. Microbiol. Immun..

